# Liposome-Based Bioassays

**DOI:** 10.3390/biology9080202

**Published:** 2020-08-01

**Authors:** Jacopo Sforzi, Lorenzo Palagi, Silvio Aime

**Affiliations:** Dipartimento di Biotecnologie Molecolari e Scienze per la Salute, Centro di Imaging Molecolare, Università degli Studi di Torino, Via Nizza 52, 10124 Torino, Italy; lorenzo.palagi@unito.it (L.P.); silvio.aime@unito.it (S.A.)

**Keywords:** liposomes, bioassays, molecular and cellular recognition, analytical chemistry, diagnostics

## Abstract

This review highlights the potential of using liposomes in bioassays. Liposomes consist of nano- or micro-sized, synthetically constructed phospholipid vesicles. Liposomes can be loaded with a number of reporting molecules that allow a dramatic amplification of the detection threshold in bioassays. Liposome-based sensors bind or react with the biological components of targets through the introduction of properly tailored vectors anchored on their external surface. The use of liposome-based formulations allows the set-up of bioassays that are rapid, sensitive, and often suitable for in-field applications. Selected applications in the field of immunoassays, as well as recognition/assessment of corona proteins, nucleic acids, exosomes, bacteria, and viruses are surveyed. The role of magnetoliposomes is also highlighted as an additional tool in the armory of liposome-based systems for bioassays.

## 1. Introduction

Liposomal vesicles are made up of a lipid bilayer surrounding the inner aqueous compartment that may be filled with solutions of molecules of interest. Hydrophobic systems can also be incorporated in liposomes as they reside between the acyl hydrocarbon chains of the vesicle membrane. Liposomes were first described by Bangham and Horne in 1961 at the Babraham Institute in Cambridge [1]. In principle, liposomes appear as the simplest mimics of a biological cell. Their biocompatibility and biodegradability have attracted much interest in the medical field, in particular as carriers for the delivery of drugs to pathological tissues [2,3]. Several liposome-based formulations have entered clinical trials and some of them are already applied in the cure of important diseases, mainly in the field of cancer therapy, but also as antibacterial and antifungal agents [4,5,6,7]. Moreover, the interaction of liposomal nanoparticles with the host immune system also plays a pivotal role in the vast and promising field of medicine and immunotherapy [8,9].

In this survey, we aim to explore the in vitro use of liposomes, either as amplification effectors or as molecular recognition agents, thanks to the functionalizations that may be introduced on their outer surface. In principle, any bioassay that is based on the detection of a molecular recognition event can be suitably amplified by replacing the reporting moiety with a vesicle containing many copies of a given censoring agent. Moreover, further amplification can be achieved when the liposomal payload is represented by an enzyme. An important step in the set-up of a liposome-based assay deals with the separation of vesicles bound to the target epitope from the unreacted forms. The detection stage often involves the release of the payload from the targeted liposome. This operation is easily pursued by the action of tension-active substances such as sodium dodecyl sulphate (SDS) or upon the stimulus of ultra-sounds or enzymes such as phospholipases [10].

For the applications designed to exploit amplification effects brought upon by the liposome payloads, it is of course relevant to know the number of reporting molecules that can be loaded into liposomes. The answer is related to the characteristics of the entrapped molecule, the size and lamellarity of the liposome, the composition of its membrane, the procedure followed in the loading process, etc. This question was carefully addressed in the case of liposomes loaded with gadolinium-containing agents for magnetic resonance imaging (MRI) applications, and there was enough consensus on numbers of the order of 10^5^ in the case of agents with a molecular weight <1 kD and liposomes with a diameter of about 100 nm [11,12,13].

However, interesting procedures addressing the problem of increasing the number of uploaded molecules have been reported. For instance, the design of generating weak acid or weak basic salts in the inner aqueous cavity has been exploited to enhance the encapsulation of various commonly used drugs (i.e., doxorubicin) and fluorescent probes [14]. In order to obtain enhanced amplification effects, beyond the simple multiplicative effect of the achievable response due to the presence of many signaling molecules delivered by the vesicles, liposomes having more recognition vectors on their external surface may allow the attainment of an improved binding to the target epitopes. This “multi-valence” effect may be particularly useful in assays involving cells, exosomes, or supramolecular systems. This field is only partially investigated but appears very promising in terms of the development of innovative liposome-based bioassays.

The peculiar properties of liposomes make them interesting systems for novel applications in biology and medicine. First of all, they can provide large amplification effects thanks to the reporter payload and they may contribute to the assessment the recognition step of the targeted analytes. Moreover, their sizes make them suitable tools for multiple interactions to enhance the binding to biological entities, from proteins to nucleic acid, to exosomes, viruses, and bacteria. The aim of this survey is to explore the main achievements in the field, in light of providing a basis for researchers to design new bioassays based on liposomes properties.

## 2. Generalities about Liposomes

When phospholipids are dissolved in water and sufficient energy is provided to the solution by sonication, heating, homogenization, or other methods, bilayered structures are formed [15]. This phenomenon is thought to be related to the critical micelle concentration (CMC), defined as the concentration of lipids in hydrophilic solvents above which lipids form vesicles or micelles, rather than remaining soluble in their monomeric form. It is thought that liposome formation in the lipid-in-water suspension originates from the bilayer fragmentation with subsequent self-closure of bilayered fragments [16]. In short, liposomes can be defined as artificial lipid-based bilayered vesicles. During the “self-closure” event, the forming liposome is able to entrap soluble molecules inside its aqueous core, a feature often exploited in both research and applied life sciences. According to their size and to the number of bilayers forming the vesicles, liposomes can be classified as (i) small unilamellar vesicles (SUV), size range 20–100 nm; (ii) large unilamellar vesicles (LUV), size range 100–1000 nm; (iii) giant unilamellar vesicles (GUV), size > 1000 nm; (iv) oligolamellar vesicles (OLV), size range 100–1000 nm; (v) multilamellar large vesicles (MLV), size range > 500 nm; (vi) multivesicular vesicles, size from 1000 nm to several thousand nanometers (see Table 1).

As far as their composition is concerned, the following phospholipids are more frequently encountered: phosphatidylcholine (PC), phosphatidylethanolamine (PE), phosphatidylserine (PS), and phosphatidylglycerol (PG) [3]. Cholesterol is also a common component that stabilizes the liposome bilayer by reducing its permeability in physiological fluids. A parameter of paramount importance in the manufacture and applications of liposomes deals with the phase transition temperature (Tt) of these phospholipids, i.e., the temperature above which phospholipids exist in liquid crystalline phase. In fact, it is in this fluid state that the mobile hydrophobic chains form the bilayers, which are the membranes of the liposomes. Lipid mixtures can have multiple transition temperatures (e.g., crystalline, gel, liquid-ordered, liquid-disordered), and the identification of the right temperature is particularly important when using multicomponent lipid mixtures. Below the Tt, the phospholipids are in the gel state, i.e., in a well packed arrangement, not ready for the formation of the bilayers. Over time, several methods have been proposed for the preparation of liposomes [17]. Important advances have been made to overcome drawbacks related to the traditional thin layer methods. These include high pressure methods [18,19] and supercritical assisted techniques [20,21,22] that have been developed in recent years.

**Figure 1:**
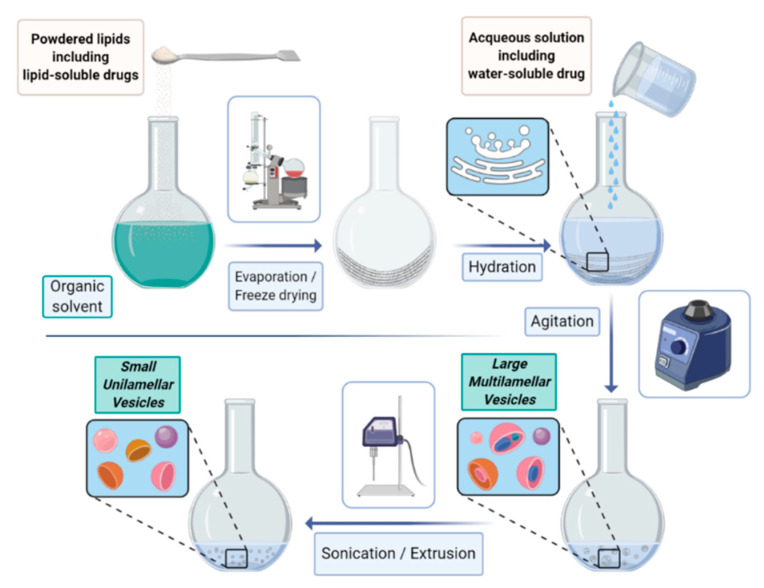
Laboratory procedure for liposome formation. A lipidic film is formed after dehydration in a rotary evaporator, and subsequently rehydrated with an aqueous solvent. The formed vesicles present different sizes and lamellarity, and are then further treated by sonication or extrusion procedures to achieve the desired liposome characteristics.

The most common procedure (schematized in Figure 1) relies on the following steps: (i) lipid dissolution in organic solvents, (ii) drying of the resultant solution, (iii) hydration of dried lipids (using various aqueous media), (iv) isolation of the liposomal vesicles, and (v) quality control tests.

By this method, lipids are firstly dissolved in an organic solvent and dried to yield a thin film at the bottom of the flask. The film is then hydrated by adding an aqueous solution (containing the compounds to be entrapped into the aqueous cavity) to yield the liposome suspension. According to the way the hydration (often under bath sonication) is carried out, SUV, GUV, or MLV are formed. The size selection is commonly achieved by a consecutive extrusion through filters of defined pore sizes.

An alternative approach to the film hydration consists of the reverse phase evaporation that relies on the sonication of the mixture of the aqueous and organic phases followed by the removal of the organic phase under reduced pressure. Once formed, liposomes are firstly characterized in terms of vesicle size, size distribution (polydispersity), lamellarity, and surface charge (zeta potential) [23]. The technique of choice is represented by dynamic light scattering (DLS) that is based on the assessment of extent of fluctuations in light intensity associated with the diffusion rate of the suspended particles. DLS also provides information on zeta potential by assessing changes in the scattered light intensity caused by particle motion due to the applied electric field. The access to the microscopic observation (TEM and even better cryo-TEM) is important as it provides the direct visualization of the individual particles with an accurate determination of size, shape, and morphology [24]. For in vivo applications, much work has been devoted to cover the liposomal surface with long polyethylene glycol (PEG)-containing chains, with the aim of masking the charges that are responsible for the recognition (and elimination from circulation) from the reticulo-endothelial system (RES) [25]. Conversely, the use of cationic or anionic liposomes is pursued in the case of applications where the charge allows for the promotion of binding to antigens or to promote the cellular uptake.

## 3. Liposome Immunoassays

**Figure 2:**
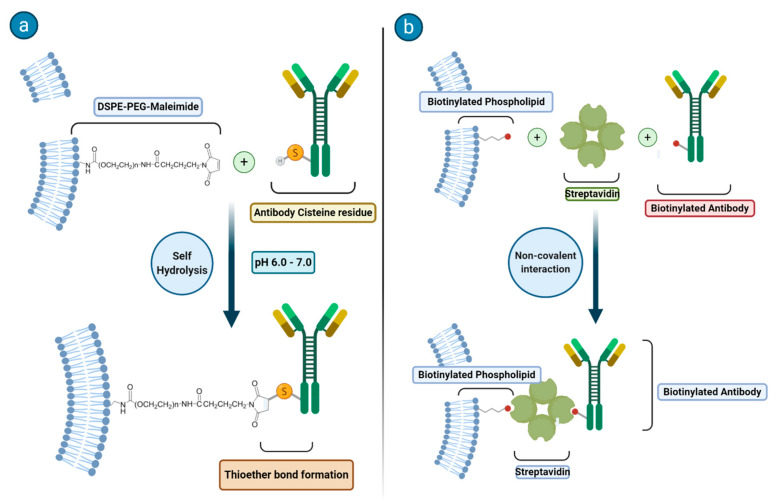
Two synthetic strategies for immunoliposome formation. (**a**) Some of the phospholipids of the lipid bylayer are functionalized with a maleimide moiety that will react with a cisteine residue present on the antibody to form a thioeter bond. (**b**) Liposomes that expose biotin residues on their surface first bind to streptavidin, which contains four high affinity biotin binding sites, and next a biotinylated antibody recognizes an untaken biotin site on the protein yielding to the formation of the antibody-functionalized liposome.

Immunoassays are biological analytical techniques in which the quantitation of an analyte relies on the specificity of the reaction between an antigen (analyte) and an antibody. The use of liposomes whose surface is functionalized with antibodies is under intense scrutiny as it appears to offer promising strategies in many diagnostic and therapeutic fields including cancer, as well as inflammatory, cardiovascular, infectious, autoimmune, and neurodegenerative diseases [26,27]. Much attention has been devoted to the synthetic methodologies related to the attachment of antibodies or other ligands to the surface of the liposomes. In Figure 2, the two common synthetic methodologies for immunoliposome formation are schematized. In principle, one may go with an anchoring step that is carried out before or after the preparation of the liposome and the targeting moiety may be covalently or noncovalently bound to the phospholipids or on the top of an additional moiety (often represented by a PEG chain).

In the case of a covalent binding, a largely applied method makes use of the reactivity of a sulfhydryl group on the protein and of a maleimide functionality (that is easily introduced on the phospholipids) to form a thioether bond. Another route that is often considered deals with the use of biotinylated phospholipids to which streptavidin can tightly bind to yield a system ready for forming an additional bond with a biotin moiety previously attached at the surface of the antibody.

Although not yet approved for clinical use, a number of studies support the view that immunoliposomes are able to target pathological sites in an analogous fashion, as shown by monoclonal antibodies or their fragments [28]. Of course, much interest deals with in vivo studies involving the development of drug delivery systems that accumulate at selected epitopes on the target cells. Furthermore, the design of the nanocarrier may include the possibility of a triggered release of its payload through a proper responsive design of the liposome characteristics. When the liposomes are loaded with imaging agents, immunoliposomes are of great interest for diagnostic applications within a molecular imaging approach. On this basis, immunoliposomes have been considered in the context of different signal amplification strategies to improve the detection sensitivity of immunoassays [29,30]. Liposomes have been considered extremely attractive materials for signal amplification because they can be loaded with a large amount of signaling molecules, leading to a high sensitivity. Thus, liposomes have then been utilized as signal enhancers in analytical biochemical assays for decades in various formats, with antibodies being the most powerful antigen recognition vectors [29,30,31].

**Figure 3:**
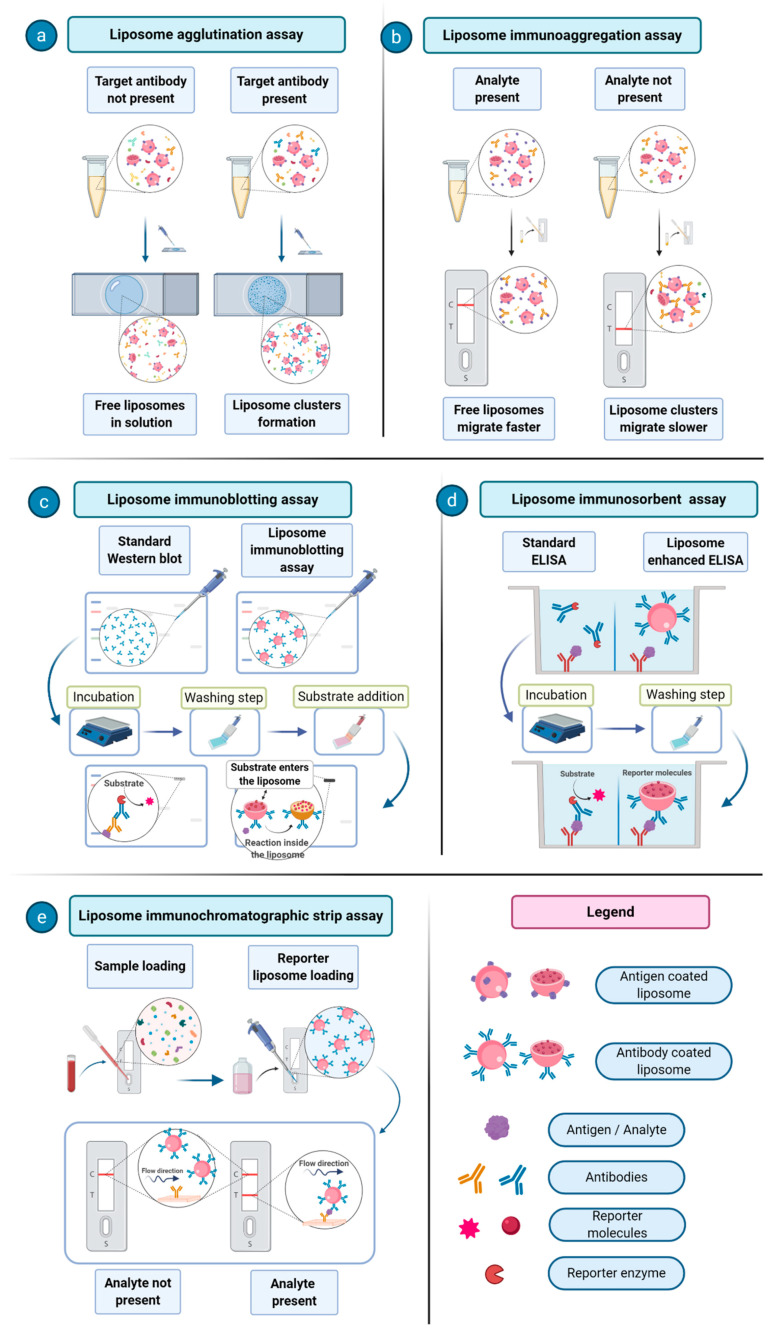
Examples of immunoliposome-involving assays. (**a**) Liposome agglutination assay: (left) liposomes do not aggregate in the absence of sample antibodies (Abs); (right) in the presence of multivalent Abs, liposomes form clusters which are visible by optical microscopy. (**b**) Liposome immunoaggregation assay: (left) in the presence of the analyte, Abs are not able to cluster liposomes into aggregates, leading to their fast migration; (right) Abs interact directly with the liposomes due to the absence of the analyte. (**c**) Liposome immunoblotting assay: protein antigens (Ags), previously separated by Western blot protocol, are probed with Ab-tagged enzyme-encapsulating liposomes. A substrate soluble into the lipid bilayer is used to obtain improved sensitivity over common enzyme Ab-conjugated reporter molecule. (**d**) Liposome immunosorbent assay: liposomes are used in the common ELISA protocol setting as signal amplifiers, compared to Abs conjugated with single reporter molecules. (**e**) Liposome immunochromatographic strip assay: liposomes are able to interact with an analyte previously captured by a primary Ab immobilized on a solid support, yielding a strong signal per analyte molecule.

In immunoassays, the liposomes are loaded with many types of signal-generating labels, such as fluorescent probes, metals, and chelates. Loading liposomes with enzymes is also a possibility, especially when a further improvement of signal amplification and sensitivity is needed [32]. In liposome immunoassays (LIA), liposome-encapsulated markers (e.g., a fluorescent dye) are prepared as phospholipid compositions coupled with either an analyte or antibody using standardized procedures [33]. An efficient example of enzyme-loaded liposomes has been described in the context of a liposome immunoblotting assay, where liposomes carrying high quantity of reporter enzymes were exploited to enhance the signal given by proteins of interest in the known Western blot procedure [34]. One of the first proposed immunoassays consisted of an agglutination assay, where liposomes carrying an antigen of interest on their surface are made to interact with antibodies against a certain pathogen in order to assess the presence of a given disease [35]. An interesting application of immunoliposome was reported for the detection of the environmental contaminants polychlorinated biphenyls (PCBs). It was based on the detection and quantification of liposome migration along strips of backed nitrocellulose. These analyte-tagged, dye-encapsulating liposomes compete with the sample PCBs for the limited number of antibody binding sites. The competitive reaction between analyte-tagged liposomes and the sample analyte for immobilized antibodies is detected through the visualization of different migration bands on a nitrocellulose support [36]. Over the years, much progress has been made in the development of immunoliposome assays in areas including immunoliposome-coupled enzyme-linked immunosorbent assay (ELISA), immunoliposome-coupled magnetic separation, and liposome-based immunochromatographic strip assay [37]. In Figure 3, a few examples of immunoliposomes-involving assays are schematized and their mechanisms are described.

**Figure 4:**
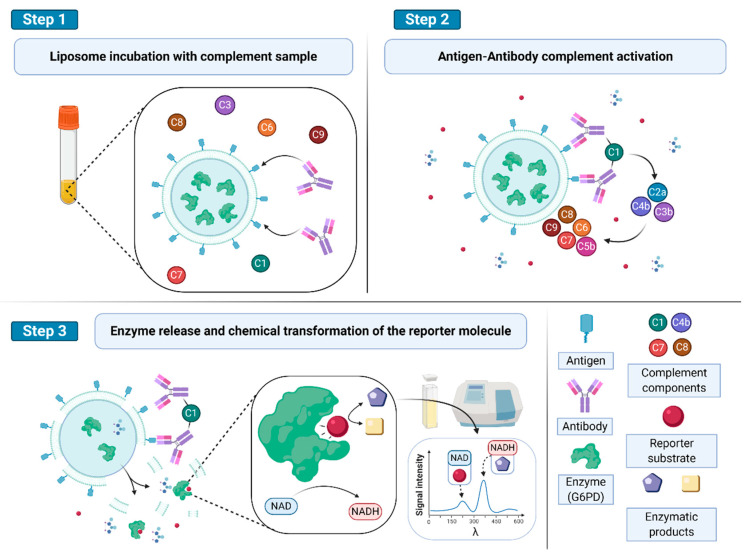
Detection of complement and antibody (Ab) activity in blood samples. (**Step 1**) Abs, added or already present in the serum sample interact with liposome-bearing specific antigens (Ags) on their surfaces. (**Step 2**) Complement is activated by the presence of the Ag–Ab interaction on the liposome surface. (**Step 3**) Complement activity disrupts or alters the phospholipidic bilayer permeability, allowing the access of the reporter substrate to the enzyme. The cleaved substrate yields a detectable signal carrying information on the complement activity. Figure inspired by [38].

An example of a typical application of liposome immunoassay is the one that was developed by Yamamoto and coworkers and commercialized by Wako Chemicals for the assessment of total complement in serum [38]. The complement cascade, consisting of 20 serum proteins, reports on the immunological defense system. Its assessment is important in the diagnosis of many diseases such as systemic lupus erythematosus, rheumatoid arthritis, cryoglobulinemia vasculitis, some forms of nephritis, and inherited deficiencies of the complement system. The method relies on the immune lysis of dinitrophenyl (DNP)-labeled liposomes, caused by complement activity and detected spectrophotometrically thanks to the activity of the entrapped enzyme, glucose-6-phosphate dehydrogenase (G6PD), on glucose-6-phospate added to the assay medium (see Figure 4).

The field of liposome-based assays has been extensively surveyed in past years and we invite the interested reader to refer to the excellent published reviews [39,40,41]. In the following paragraphs, we focus on novel approaches that have reported on the detection/amplification effects achieved with the use of liposomes in the field of corona proteins; in the detection/assessment of nucleic acids; and in the recognition of exosomes, viruses, and bacteria.

## 4. Liposomes Binding to Protein Corona

**Figure 5:**
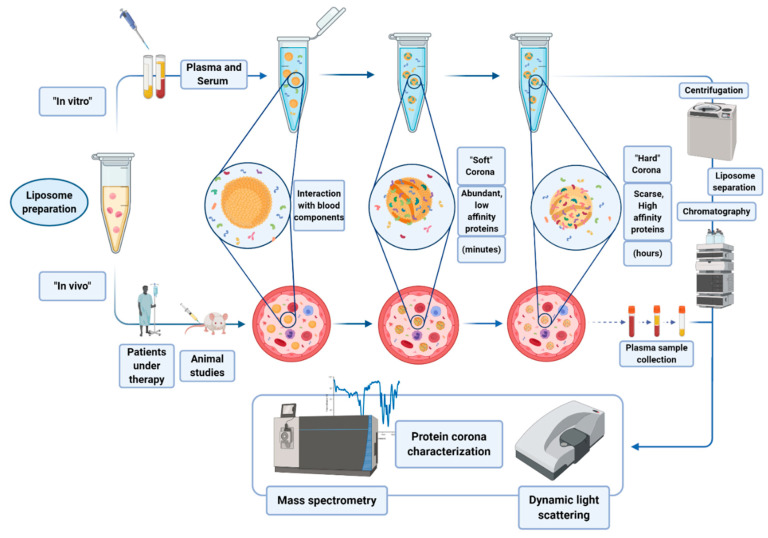
Protein corona formation and analysis.

Protein corona refers to the complex of proteins and other molecules that are involved in the interaction to nanoparticles (NPs) in biological fluids. Molecules are adsorbed on the NP surface on the basis of the chemical characteristics of the NP itself. Usually, a first layer of proteins abundant in the matrix interacts with the NP surface, forming the so called “soft” corona. Such a layer may then be substituted by the “hard” corona, made of less abundant protein, but with a higher affinity for the NP (see Figure 5) [42].

Protein corona can be formed both in vitro, incubating the nanoparticles (NPs) with biological samples, or directly in vivo. Hajipour et al. reported that various types of disease can specifically affect the composition profile of protein corona [43]. These findings have been exploited by different research groups by using liposomes as NPs able to scavenge the blood pool and surface-capture low-molecular-weight and low-abundance plasma proteins that cannot be detected by conventional plasma proteomic analysis. During the last decade, protein corona research has gained popularity, with many attempts made to molecularly characterize corona profiles after the ex vivo incubation of NPs in biofluids (mainly plasma), and more recently, upon allowing their in vivo circulation in rodents [44,45,46,47]. Hadjidemetriou et al. analyzed and characterized the protein corona formed onto polyethylene glycol (PEG)ylated doxorubicin-encapsulated liposomes (Caelyx), recovered from the blood of ovarian carcinoma patients [48]. Their study described the protein corona formed on the outer liposome surface and highlighted the potential of such a novel tool in accessing the blood circulation proteome. An interesting work was carried out by Palchetti and coworkers, who studied liposomes uptake and drug formulations in pancreatic ductal adenocarcinoma patients [49]. In this context, the emerging concept was that the limited success of liposome-delivered drugs in clinical practice is due to the poor knowledge of the nano–biointeractions experienced by liposomes in vivo. Recent studies have shown that only a minor fraction of the hundreds of bound plasma proteins, referred to as “phosphatidylcholine (PC) fingerprints” (PCFs), enhance liposome association with cancer cells, triggering efficient particle internalization. In their work, they synthesized 10 different liposomal formulations with systematic changes in lipid composition and exposed them to human plasma (HP) [50]. Through dynamic light scattering (DLS), micro-electrophoresis, and nano-liquid chromatography–tandem mass spectrometry (nano-LC–MS/MS), they were able to characterize the different corona protein adsorbed on the liposome surfaces, leading to a better understanding of the interactions that drive the uptake of liposomes into the cancer cells of interest with less collateral effect. Moreover, they found that some of the studied NPs are enriched with plasma proteins that are associated with the onset and progression of pancreatic ductal adenocarcinoma (e.g., sex hormone-binding globulin, ficolin-3, plasma protease C1 inhibitor, etc.). Such ability of liposomes to trap molecules on their surface could open the intriguing possibility of identifying novel biomarkers. Furthermore, combining the concepts of disease-specific protein corona and sensor array technology, Caracciolo et al. developed a platform with disease detection capacity using blood plasma samples obtained from patients diagnosed with five different cancer types (i.e., lung cancer, glioblastoma, meningioma, myeloma, and pancreatic cancer) and a control group of healthy donors. Exploiting three different liposome formulations, with distinct lipid composition and surface charge, they showed that distinct patterns of the protein corona composition revealed a unique ‘‘fingerprint’’ for each cancer type [51].

## 5. Liposome-Based Nucleic Acid Detection Assays

Nucleic acids are not only biological molecules with a unique and crucial role in biology, but they are also molecules able to support peculiar applications in the analytical chemistry field. Nucleic acids can be detected with the well-established PCR methods, pivotal procedures in any biological laboratory, greatly improved in the last decades. The analysis of nucleic acids through lateral flow or sandwich hybridization assays carries the possibility to detect RNA or DNA molecules without amplification procedures and in a short time [52]. Moreover, interesting applications involving liposomes in the detection of nucleic acids appear worthy of consideration. Different techniques exploiting liposome signal amplification have been developed and improved during the past three decades, leading to increased interest in the field of analytical chemistry for the development of assays in the diagnostic, food, and environmental science fields [53].

**Figure 6:**
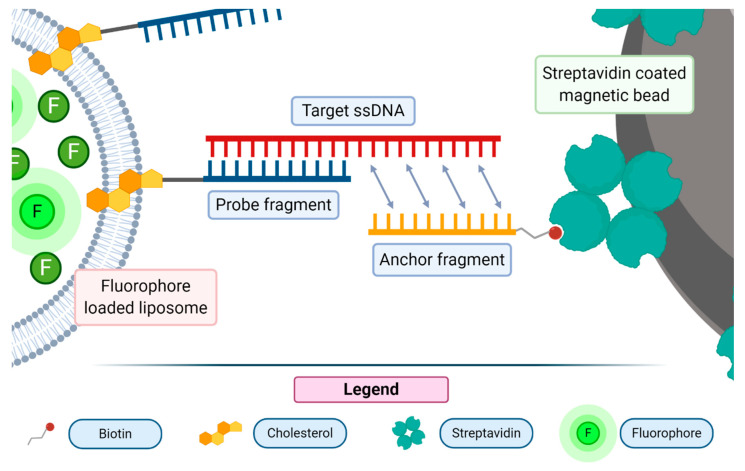
Sandwich hybridization assay: an anchored single-stranded DNA probe is used to selectively and specifically interact with the nucleic acid sequence of interest. Dye encapsulating liposomes, carrying on their surfaces a second probe, complementary to the second portion of the target nucleic acid, are responsible for the anchoring step, yielding the “sandwich” type association. Upon washing out the unbound liposomes, the bound ones are quantitatively assessed upon the release of their fluorescent payload. Figure inspired by [57].

One of the methods used for the detection of nucleic acids consists in the sandwich hybridization assay, a technique where DNA probes are used to selectively bind to a target sequence of interest, firstly described by Dunn and Hassell in 1977 [54]. The probe fragments can be directly anchored to a solid support by covalent bonds or by the non-covalent interaction of biotinylated oligonucleotides and streptavidin-coated plates or beads. For this application, the liposomes are usually loaded with fluorescent molecules. The sandwich hybridization assay has been used to detect a wide array of nucleic acid molecules, from RNA (messenger RNA, micro RNA, ribosomal RNA) to single-stranded DNA [55,56]. In such assays, the strong liposome signal enhancement feature may be exploited to report about the recognition of a nucleic acid probe. The most used dyes encapsulated in liposomes are sulforhodamine and carboxyfluorescein [53]. One of the most recently proposed assays of this class used 18 bp long probes bound to streptavidin-coated magnetic beads through a biotin moiety [57]. Liposomes were previously conjugated with a DNA probe by means of a cholesterol moiety, able to intercalate in the phospholipid bilayer of the vesicle. The exposed single strand DNA moiety, upon the recognition of the bound fragment, anchored the liposome-containing probe to the solid support (see Figure 6).

The unbound liposomes were then washed away and the bound ones were disrupted with a detergent molecule (Triton-X100). The released fluorescent dye was then analyzed with a spectrofluorometer. This assay displayed a good sensitivity as it resulted in the ability to detect single-stranded DNA (ssDNA) down to the concentration of 7 × 10^−13^ M. Improvements are ongoing to pursue the detection of RNA molecules with a further increase of the assay sensitivity.

**Figure 7:**
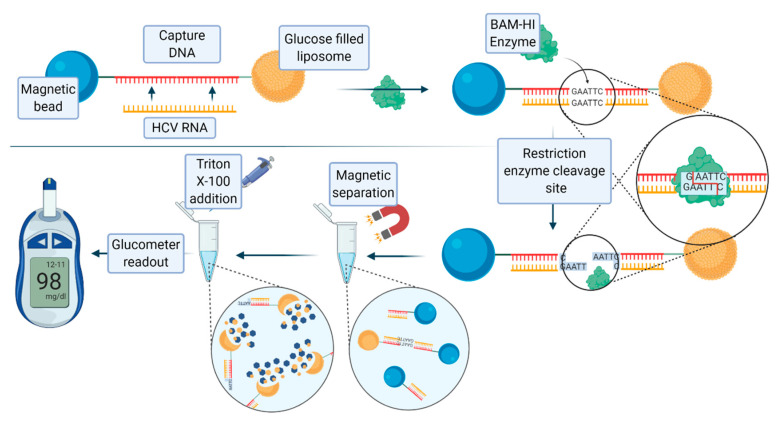
Detection of hepatitis C virus (HCV) RNA through the interaction of glucose-loaded liposomes with probe carrying magnetic beads. Upon the action of the restriction enzyme BamHI, the release of the glucose-containing liposomes occurs. Next, the release of glucose from the liposomes takes place by the addition of Triton-X-100 and is determined through a glucometer. Figure inspired by [60].

The unique properties of nucleic acids inspired new approaches for the recognition of specific analytes. For instance, aptamers, a class of nucleic acid mimics with a singular three-dimensional structure, have been considered as high affinity binders for a given ligand, thus paving the way for their use in the detection of different molecules [58]. Instead of using antibody-coated liposomes, aptamers have been used both on solid supports and on reporter lipid nanoparticles, often filled with fluorescent molecules. An interesting application dealt with the detection of cholera toxin [59]. A recently published nucleic acid assay for the detection of hepatitis C virus RNA, developed by Tu et al., exploited the use of glucose-loaded liposomes, magnetic beads, and the endonuclease BamHI [60]. A 21 base pair long oligonucleotide complementary to hepatitis C virus (HCV) RNA was covalently conjugated to a magnetic bead, and then bound to the glucose-loaded liposome. When the target HCV RNA is present, this RNA strand hybridizes with the oligonucleotide to form double-stranded RNA–DNA heteroduplex. The enzyme BAmHI is then able to cleave the double strand molecule at the specific 5′-GGATCC-3’, releasing the liposome from the magnetic bead. Following magnetic separation, the glucose-loaded liposomes are lysed with Triton-X-100 to release the payload of glucose molecules, which are then detected with a digital portable personal glucometer (PGM). Figure 7 shows the scheme and description of this assay. The limit of detection (LOD) of the system was 1.9 pM. Human serum samples containing HCV RNA were analyzed using this method, yielding promising results.

**Figure 8:**
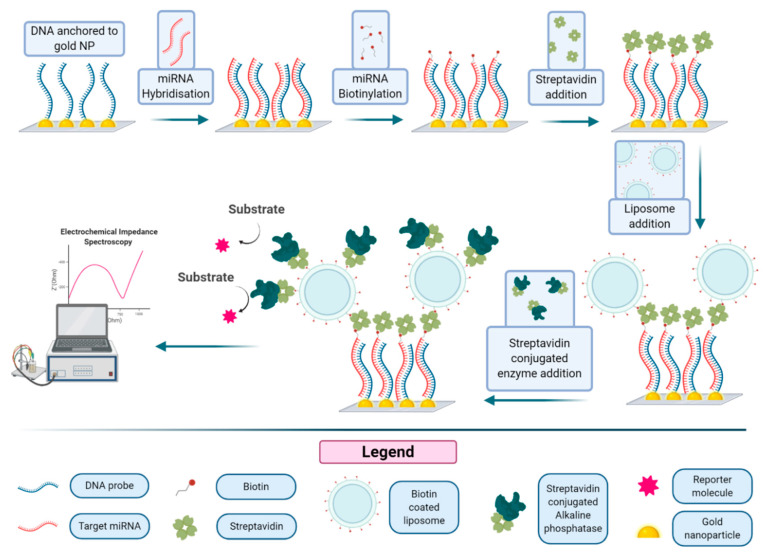
Biotinylated liposome amplification for the detection of micro-RNA: single-stranded DNA probes are bound to the surface of gold nanoparticles, and made to interact with the miRNA of interest, which is subsequently biotinylated. A biotinylated liposome is then anchored to the miRNA through a streptavidin molecule, and further recognized by streptavidin-tagged enzyme. The signal obtained by the enzyme–substrate interaction is then quantified. Figure inspired by [61].

A related approach, illustrated in Figure 8, based on the immobilization of thiolated DNA capture probes onto gold nanostructured carbon surfaces, was reported for the detection of micro RNA using liposomes decorated with streptavidin alkaline phosphatase [61]. Gold nanoclusters and probe DNA are immobilized on a nanostructured surface. This capture probe-modified Au-SPCE (screen-printed three-electrode strip based on a carbon working electrode), backfilled with mercaptohexanol, was incubated with the micro-RNA-222, a micro-RNA that is found in serum and overexpressed in some cancers. The miRNA molecule can be biotinylated under specific conditions, leading to the formation of a hybrid on the Au-SPCE surface, due to the interaction with the DNA probe. Then streptavidin and biotinylated liposomes are added, forming a structure that is finally decorated with streptavidin alkaline phosphatase. After incubation of the substrate, the enzymatic product is revealed by electrochemical impedance spectroscopy (EIS). The limit of detection of this amplification method was reported to be 400 fM, with a limit of quantification of 1.70 pM, 20 times lower than that obtained using a simple enzyme conjugate for the detection. Application of the optimized assay in serum samples was also demonstrated.

**Figure 9:**
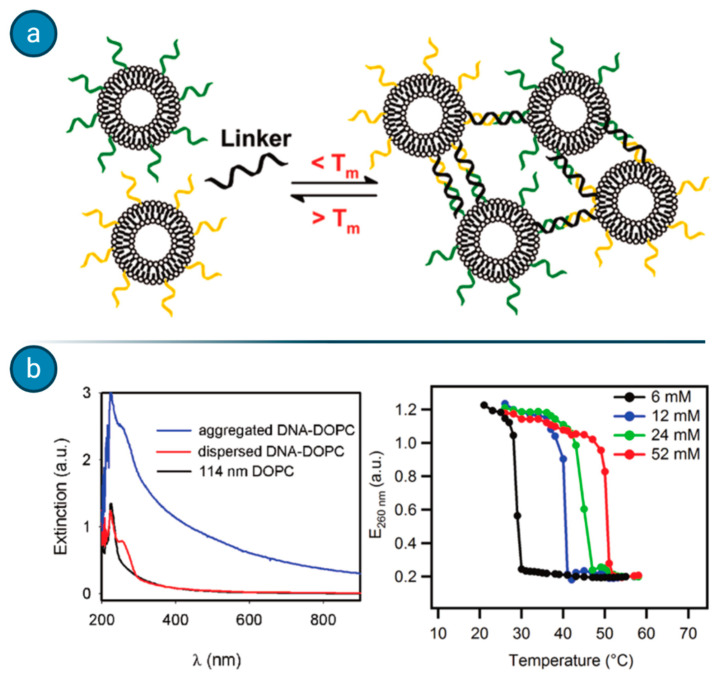
Single-stranded DNA-mediated liposome aggregation assay. (**a**) In the presence of a target nucleic acid (below the respective melting temperature, T_m_), liposomes bearing specific probes on their surfaces are able to interact with the analyte and form clusters. (**b**) Cluster presence is then determined spectrophotometrically. Figure adapted with permission from [62]. Copyright 2011 American Chemical Society.

Agglutination assays, which rely on the formations of liposome clusters when the target analyte is present, have been exploited by Dave and Liu for the detection of ssDNA [62]. When liposomes are coated with DNA probes and incubated with the fragment of interest, a cluster of different liposomes forms, switching the UV–VIS spectra absorbance of the compound (see Figure 9). Such a simple and fast method could be exploited for the detection of different nucleic acids as well for other macromolecular analytes, although the major issue appears to be the sensitivity with the relatively high detection threshold.

## 6. Magnetoliposomes

In the last 3–4 decades, magnetoliposomes have found increasing interest in an array of biomedical applications such as tissue-specific drug delivery for cancer therapy, gene delivery, multifunctional medical imaging, hyperthermia treatment, and diagnostics. The term “magnetoliposome” was first coined in 1988 by De Cuyper and Joniau to describe magnetic nanoparticle-coated liposomes, but it may also refer to encapsulated solutions of ferro- or paramagnetic materials within the aqueous core, as well as hydrophobic magnetic species incorporated into the phospholipidic bilayer [63]. The properties of magnetoliposomes vary significantly with their composition and can be tuned through choosing the appropriate magnetic species and phospholipidic composition of the bilayers. In principle, the interest for magnetoliposomes is twofold, i.e., either for their role as signal amplifier and for the possibility of exploiting the magnetic attraction in separation-guided procedures.

Most magnetoliposome in vivo applications concern their use as drug delivery platforms and medical imaging agents, due to their capability of being guided through a magnetic field to a specific target site and their ability to generate a detectable signal. Thus, many studies of magnetoliposomes designed for encapsulating and releasing drugs can be found in the literature, as well as in terms of their application in multi-modal imaging, combining the potential of magnetic resonance imaging (MRI), fluorescence spectroscopy, positron emission tomography (PET), and other imaging modalities. Findings from drug delivery and medical imaging applications may be exploited for translation to the in vitro diagnostics realm. The translation from in vivo to in vitro applications has first of all dealt with the iron oxide-containing liposomes (i.e., either in the inner aqueous core or incorporated in the lipidic bilayer), as these systems have been under intense scrutiny in in vivo diagnostic applications [64].

**Figure 10:**
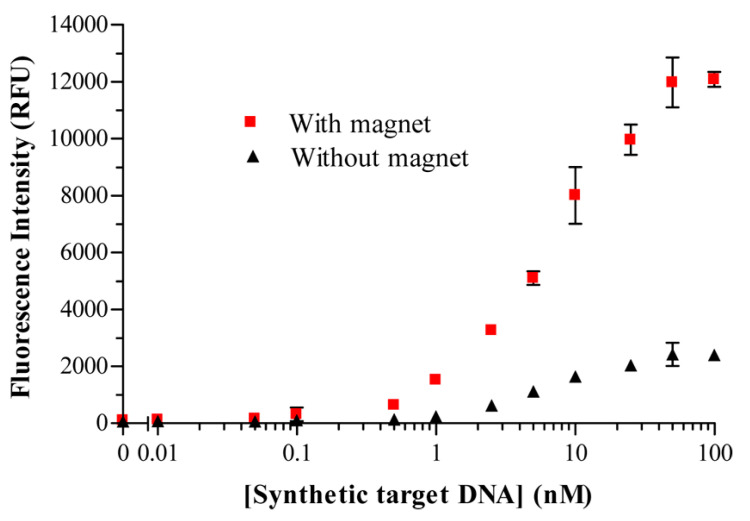
Sensitivity and limit of detection (LOD) enhancement in the presence (squares) versus absence (triangles) of a magnet. Figure reprinted with permission from [65]. Copyright 2014 American Chemical Society.

A typical example that outlines the advantages associated to the use of magnetoliposomes in bioassays is the one reported by Edwards and Baeumner [41]. They tackled the drawback that accompanies any heterogeneous binding assays, which deals with the interferences between solution phase analytes and surface immobilized biorecognition elements (BREs), often leading to mass transfer limitations. They showed that an applied magnetic field can be used to promote target-binding events when the species used for signal generation is rendered magnetic, thus improving the detection threshold and decreasing the assay time. Marker-encapsulating BRE-tagged magnetoliposomes were designed for signal enhancement in analytical assays. A ferromagnetic metal oxide–oleic acid complex incorporated within the liposomal bilayer allowed for their separation under a magnetic field while maintaining the integrity of the inner cavity volume for the encapsulation of fluorescent signaling molecules. In fact, when the separation step could take advantage of the use of an underlying magnet, the DNA-tagged liposomes yielded enhanced sensitivity (see Figure 10), in addition to reduced assay times and reagent concentrations.

**Figure 11:**
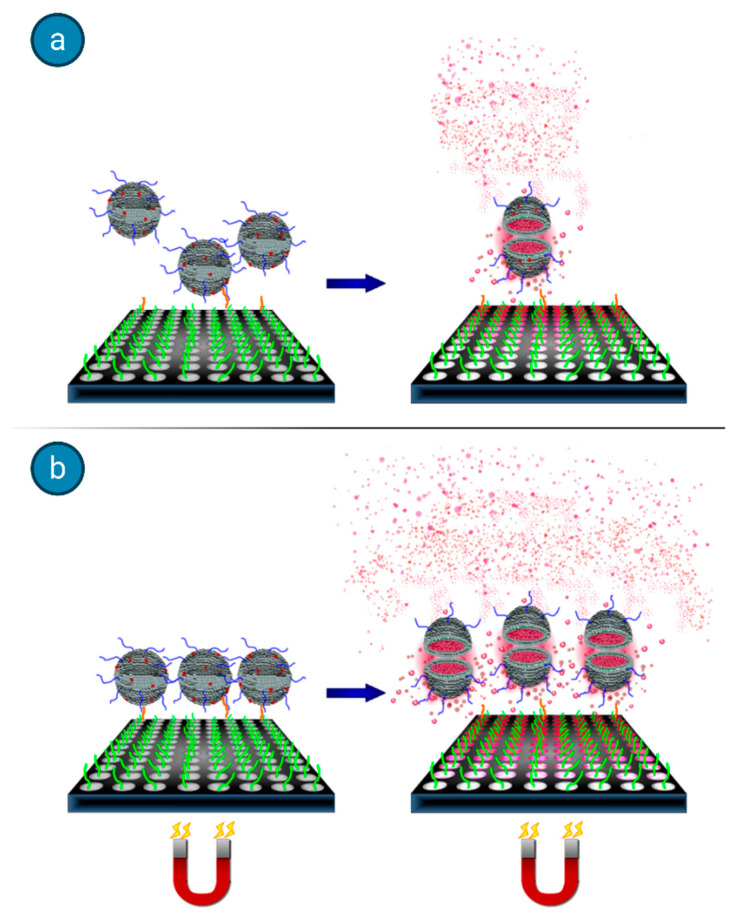
Sandwich hybridization assay with DNA reporter probe-labeled fluorescent dye-encapsulating magnetoliposomes for the quantification of target nucleic acids. (**a**) The analyte is captured on the surface, but the interactions are subject to mass transfer limitations. (**b**) When an underlying magnetic field is applied, additional binding interactions with the immobilized target are promoted, leading to increased assay sensitivity and reduced assay times and reagent concentrations. Figure adapted with permission from [65]. Copyright 2014 American Chemical Society.

In this high-throughput sandwich-hybridization assay, the magnetic signaling reagents appear to offer superior performance and adaptability to standard assay formats. Figure 11 displays how, in the absence of a magnet, the sandwich hybridization complexes form but are subject to diffusion limitations, while the presence of the magnet induces the liposomes to enhanced interactions with the captured target.

**Figure 12:**
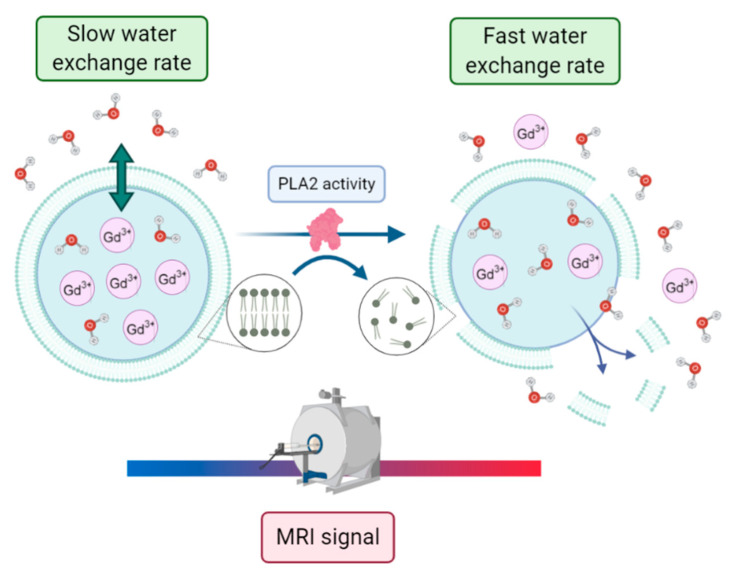
The enzyme phospholipase A2 (PLA2) catalyzes the lipidic bilayer hydrolysis of liposomes loaded with high concentrations of paramagnetic species. The water exchange rate across the liposomal membrane is increased, leading to a detectable increase in the bulk water magnetic resonance imaging (MRI) signal that is proportional to PLA2 activity. Figure inspired by [66].

Liposomes encapsulating paramagnetic agents have been shown to be useful disease-specific markers in homogeneous assays. Such assays rely on the measurement of the longitudinal relaxation time T_1_, a parameter easily accessible in any NMR experiment. Here, the determinant is often represented by the permeability of the liposomal membrane to the solvent water molecules. In fact, only in the presence of a fast exchange is the full relaxivity potential expressed, whereas in the presence of slow exchange, the observed relaxivity is largely “quenched” (the relaxivity reports on the proton relaxation rate enhancement of the “bulk” water resonance at 1 mM concentration of the agent and is expressed in units of mM^−1^s^−1^). Otherwise, one may reach the condition of maximum relaxation enhancement upon the partial or total disruption of the membrane with the release of the paramagnetic payload. In this context, Cheng and Tsourkas developed an activatable T_1_ MRI liposome-based contrast agent to monitor the phospholipase A2 (PLA2) activity [66]. PLA2s are enzymes that specifically catalyze the hydrolysis of glycerophospholipids, with changes in their activity being potentially associated with pathological conditions such as atherosclerosis, pancreatitis, and cancers. In this work, a clinically approved gadolinium-based MRI contrast agent, gadoteridol, was encapsulated within nanometer-sized liposomes. Without enzyme activity, the encapsulated paramagnetic complex showed low relaxivity due to the low permeability of the liposomal membrane to water molecules. Conversely, when PLA2 catalyzed the hydrolysis of the phospholipids within the liposomal bilayer, the complex was released into bulk solution, leading to a measurable increase in relaxivity (see Figure 12).

Such magnetoliposomes loaded with activatable MRI contrast agents can then act as nano-sensors for monitoring PLA2 activity in complex biological samples with minimal sample preparation.

A closely related approach was reported by Alberti et al. who showed that a quantitative measurement of folate receptors (FRs) on the outer cell membrane is possible by means of the relaxometric assessment of the increase in water proton relaxation rate upon the release of gadolinium-containing agents from a magnetoliposome [67]. The method consists in binding a PLA2-functionalized, folate-targeting vector to FRs (often overexpressed in tumor cells). Once the cells are labelled with the PLA2 enzyme and the unbound folate-targeting agent is removed from the cell suspension, one proceeds with the addition of an aliquot of liposomes loaded with Gd(III) complexes. The liposomal membrane was properly designed for hampering the water exchange between the inner and the outer compartment (NMR silent magnetoliposomes). Upon the action of PLA2, the liposomal phospholipids are hydrolyzed with the consequent disruption of the bilayer and, thus, the release of the paramagnetic payload. The measurement of T_1_ reports on the PLA2 activity and, in turn, on the number of the FRs on the tumor cells. The use of paramagnetic- rather than the more common fluorophore-encapsulating liposomes for measuring biomarkers activities allows interferences typical to optical methods, such as quenching and scattering, to be avoided.

**Figure 13:**
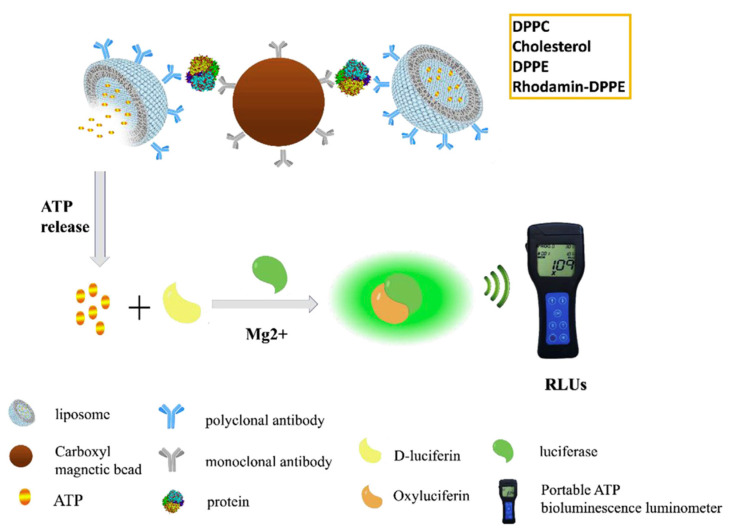
Schematic representation of the liposome-based bioluminescent magnetic (LBM) assay based on the selective capture of a given protein using magnetic beads and ATP-containing liposomes properly functionalized with the specific antibody for the protein of interest. Figure reprinted from [68], Copyright (2018), with permission from Elsevier.

Combination of antibody-functionalized liposomes and magnetic beads provides an efficient approach for the assessment of proteins of diagnostic interest. In a recent study, Liu and coworkers reported on the conjugation between bioluminescent ATP-encapsulated liposomes and magnetic nanoparticles (MNPs), both bound to antibodies able to capture the target protein [68]. These magnetic/bioluminescent liposomes containing aggregates were used in combination with a portable ATP luminometer for the rapid and sensitive detection of target protein biomarkers in blood. Alpha-fetoprotein (AFP), a biomarker associated with hepatocellular carcinoma, was used as a model protein for evaluating the method. In the presence of AFP, the complex of MNP–AFP liposomes was formed and easily isolated. The release of ATP, proportional to the AFP concentration, was measured through a portable ATP bioluminescence detector. This liposome-based bioluminescent magnetic (LBM) assay is schematically illustrated in Figure 13. Here, the liposomes play a crucial role in achieving signal amplification and transduction, providing a stable microenvironment for the encapsulated species, maximizing their activity and shielding them from destructive processes.

The output was reported as RLUs (relative light units), where 1 RLU corresponded to 1 fmol of ATP. RLUs showed a linear correlation with AFP concentration between 0.05 and 1000 ng/mL, with a limit of detection of 0.016 ng/mL. The assay exhibited high specificity for AFP, with no cross-reactivity with five other proteins tested at 25 ng/mL. A value higher than 300 RLUs indicated an AFP concentration greater than 25 ng/mL, suggestive of the possible occurrence of hepatocellular carcinoma and the need for further clinical examination. The LBM assay showed high reproducibility; accuracy; low-cost potential; and, in principle, it can be extended to a variety of proteins by replacing the antibody pairs according to the desired targets. Such innovative approach that allows for the quantitative detection of biomarkers with a portable luminometer could likely find a wide applicability in the field of the analyses of clinical samples.

In summary, although to date the attention on magnetoliposomes has been mainly focused on imaging studies or drug delivery applications, one may foresee an important role for these systems in the design of innovative bioassays. They own key features in terms of separation efficiency and signal amplification for their use as tools in bioanalysis, namely, (i) they show a large flexibility in composition, size, and MNP surface chemistry; (ii) they display a good overall stability during storage and magnetic action; and (iii) they provide an efficient bioassay performance with short times and relatively low cost [69].

## 7. Liposomes for the Detection of Extracellular Vesicles

Characterization of small extracellular vesicles (sEVs) or exosomes, circulating in body fluids, holds a great potential in the field of non-invasive diagnosis of diseases and in the evaluation of therapeutic treatments. They may be considered a novel class of biomarkers and are under intense investigation for the determination of their inner content as well as their membrane proteins for the information they may bring in terms of disease diagnosis and, more in general, on their biological functions [70,71]. However, the detection of sEVs remains a challenge due to the lack of cost-effective analytical protocols [72].

**Figure 14:**
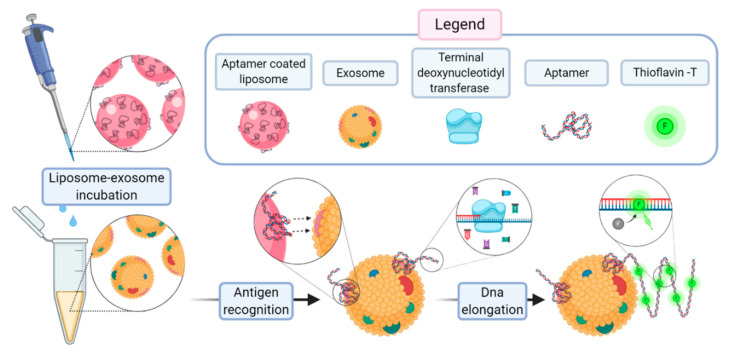
Schematic representation of the key steps of the assay based on the use of aptamer-coated liposomes. The exosome binds through its CD63 moiety (a proteic exosome marker) and the terminal deoxynucleotidyl transferase (TdT) promotes the polymerization with the inclusion of thioflavin T that is the detected reporters. Figure inspired by [73].

In a recent work, Wang et al. proposed a method for a sensitive detection of exosomes based on aptamer-coated liposome complexes coupled with the use of terminal deoxynucleotidyl transferase (TdT)-mediated polymerization [73]. In the presence of target exosomes, the aptamers immobilized on the surface of DOTAP liposomes binds to CD63 (a membrane protein exclusively found in exosomes). The resulting aptamer–exosome complex will be accessible to TdT that proceeds to the polymerization reaction. The signal amplification is achieved upon the presence in the incubation medium of thioflavin T. Figure 14 reports the scheme of the assay.

This method can be employed to profile different exosomal membrane proteins using an array of corresponding aptamers to obtain a fingerprint map of various exosomes. The authors claim a limit of detection of 360 particle/µL, corresponding to 2.16 × 10^−14^ M. With Wang’s work, an uncommon advantage of aptamer-coated liposomes over immunoliposomes became evident, as it showed the enzymatic amplification of the signal through the lengthening of the aptamer chain operated by TdT.

**Figure 15:**
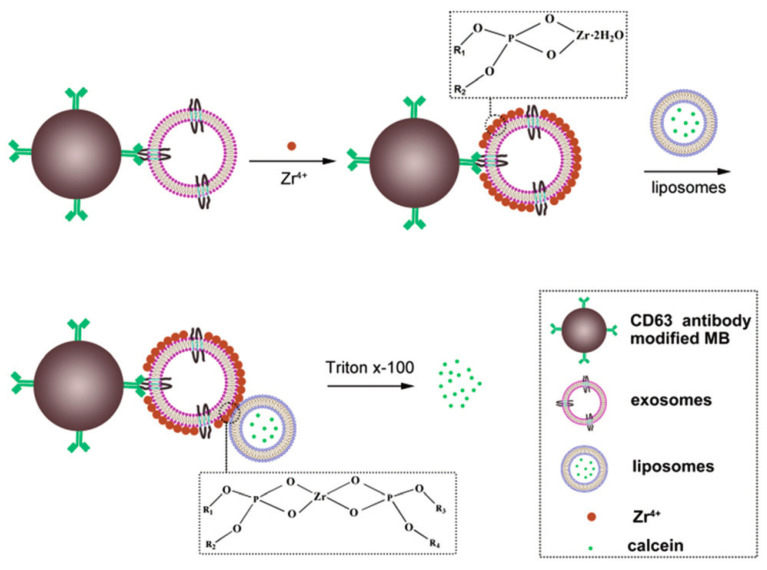
Schematic representation of the assay based on the aggregation of calcein-containing liposomes and exosomes bound to CD63-functionalized magnetic particles. Exosomes captured with antibody-coated magnetic beads are incubated with Zr4+ ions, which cover the surface of the vesicle. Liposomes are then able to link to the exosomes through the Zr4+ ions. The unbound liposomes are washed out and the bound ones disrupted by the detergent Triton-X-100. The released calcein dye is subsequently quantified by a spectrofluorometer. Figure reproduced from [74] with permission from The Royal Society of Chemistry.

Another sensitive, label-free, and cost-effective method for the detection of exosomes was proposed by the same research group through the exploitation of phosphate–Zr4+–phosphate interaction [74]. For the separation step, the proposed assay takes advantage of magnetic beads coated with antibodies against CD63, whereas for the signal amplification step, the calcein liposomal payload is used (see Figure 15). This is the first report where exosomes were detected by linking phospholipids exposed in both exosomes and liposomes with Zr^4+^ ions. The rational exploitation of the coordination capabilities of the membrane phospholipids makes the assay simpler as it does not require the introduction of extra modification in the process of target recognition and signal amplification.

The proposed method uses, as above, CD63 Ab-modified magnetic beads that bind the exosomes whose exposed phospholipids become the seeds for promoting the aggregation with calcein-loaded liposomes through the bridge provided by the Zr^4+^ ions. The limit of detection of the assay was determined to be 7.6 × 10^3^ particles/µL, equivalent to 4.57 × 10^−13^ M.

Xu et al. reported a method based on size exclusion chromatography with fluorescence detection (SEC-FD) for sEV quantification [75]. They exploited the lipophilic CM-Dil dye, a dye commonly used for cell labeling, to fluorescently label sEVs. After the incubation and separation on Sepharose CL-4B, the fluorescence of the eluent was monitored at Ex 553 nm/Em 570 nm. In this case, the liposomes (100 nm) are not directly used as the species responsible for the recognition of the analyte, rather they are exploited to differentiate the chromatographic behavior of the smaller extracellular vesicles with respect to the larger adducts made by liposomes/exosomes/magnetic particles as well as from free FITC dye. Detection limit was estimated as 2.9 × 10^7^ exosome particles/mL, corresponding to 1.56 × 10^−15^ M.

Similarly, Simonsen and coworkers exploited liposomes to calibrate their fluorescence-activated nanoparticle sorting (FANS) technique [76]. It was found that silica and plastic beads used in these procedures were unable to accurately correlate scatter intensity to the vesicle size. Therefore, a novel method to estimate the size of individual liposomes in flow cytometry was proposed, providing a kind of “calibrator” that worked well for the determination of sizes, structures, and refractive indexes of the investigated particles.

To detect extracellular vesicles (EVs), Kim and Lee fabricated a liposomal biosensor based on polydiacetylene (PDA), a conjugate polymer widely used in sensing applications [77]. To confer selectivity and sensitivity to the sensory material, antibodies targeting CD63 were attached to the PDA liposomes and phospholipid molecules were incorporated into the PDA vesicles in order to increase the nanoparticle optical properties. After incubation of the two components of the assay (i.e., PDA and EVs), signal analysis was performed by observing colorimetric changes triggered by the ligand–receptor interaction of PDA vesicles. The obtained signals from the PDA lipid immunosensor achieved a detection limit of 3 × 10^8^ vesicles/mL, equivalent to 1.8 × 10^−11^ M.

**Figure 16:**
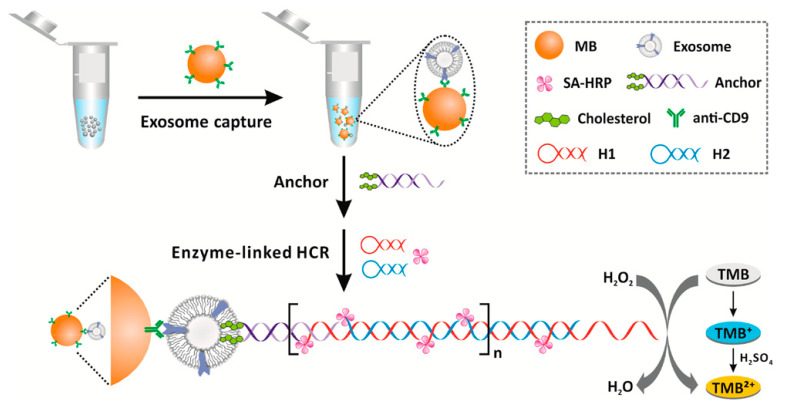
By exploiting the analogy between liposomes and extracellular vesicles (EVs), cholesterol DNA moieties are introduced on the EV membrane. Then, biotinylated DNA is introduced to provide the anchoring of streptavidin–horseradish peroxidase (HRP) for the set-up of the colorimetric determination. Figure reprinted with permission from [78]. Copyright 2017 American Chemical Society.

Cholesterol-conjugated DNA have not only been exploited for the detection of nucleic acids, but also for the detection of exosomes. As stated above, exosomes are vesicles composed of cellular components sharing a good similarity with liposomes in terms of size and membrane composition. Using cholesterol-functionalized DNA, He and coworkers designed a magnetic bead (MB)-DNA hybrid for exosome capture [78]. The MB, interacting with the exosome through the CD-9 protein, were retrieved and incubated with the cholesterol DNA. Then, a duplex DNA with a sticky end was attached to the exosomes using a bivalent cholesterol anchor. In this assay, the exosome acts similarly as a liposome, and the presence of the vesicle is detected through a hybridization chain reaction (HCR), initiated using biotinylated DNA strands, and followed by direct conjugation of streptavidin–horseradish peroxidase (HRP). The HRP catalyzed the oxidation of 3,3′,5,5′-tetramethyl benzidine (TMB), providing a colorimetric signal for the exosome determination. This assay showed a detection limit of 2.2 × 10^3^ exosomes/μL, which was 100-fold higher than ELISA-based detection mechanisms at the time. The assay is schematized in Figure 16.

## 8. Liposome-Based Assays for the Detection of Proteins, Bacteria, and Viruses

**Figure 17:**
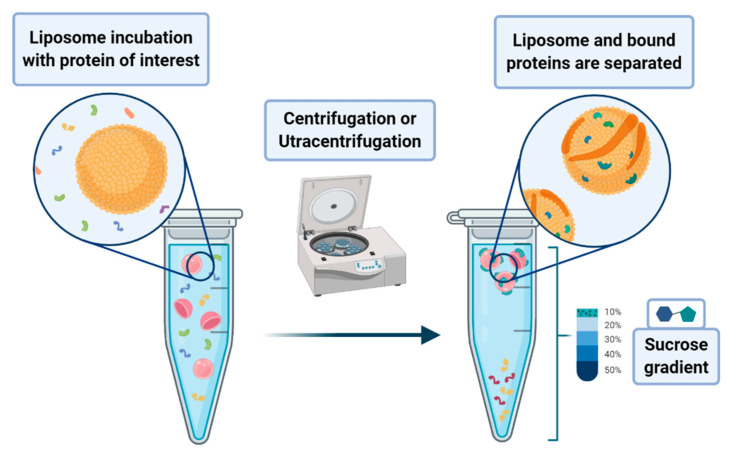
Scheme of the liposome flotation assay. Liposomes are incubated with the proteins of interest to investigate the possible interaction of the analyte with the phospholipidic membranes. Liposomes are then separated by centrifugation or ultracentrifugation, usually in the presence of gradients. The presence of the protein in the liposome specimen suggests the occurrence of a protein–membrane interaction.

A peculiar application of liposomes deals with their use to pursue the co-sedimentation or the co-flotation of proteins and other systems. This consists in adding liposomes to biological fluids containing the macromolecules one aims to separate from the solution. The process that leads to the phase separation relies on the multiplex interactions that the liposome can provide to the substrates of interest. Although it is a procedure endowed with low specificity, it can be nevertheless classified as an amplification procedure. In principle, one may envisage the possibility of enhancing the specificity of the method by designing liposomes that bear on their surface proper functionalities that act as specific recognition/anchoring sites for given molecular synthons. Much attention has been devoted to the application of these methods to the separation of proteins that are interacting with membranes, such as the intracellular layer of the plasmatic membrane or those of the organelles. In a lipid flotation assay, vesicle-bound proteins are separated from unbound proteins and vesicles alone, upon flotation of the vesicles on a sucrose gradient (see Figure 17) [79,80].

Particular attention has been devoted to phosphoinositides that are membrane lipids involved in the control of important cellular signaling and trafficking processes [81]. This occurs through the recruitment of specific effector proteins to the cytosolic face of plasma membrane and organelles. The assessment of these effector-binding properties and specificity towards different phosphoinositides is a fundamental information for the understanding of their cellular functions [82]. The flotation method was applied to assess membrane binding and insertion of hemolysin of *Escherichia coli*, a toxin that subverts host cell functions and causes cell lysis. Using properly labelled photoactivatable probes incorporated into the target lipid bilayer, it was possible to acquire relevant information about the binding mechanism between the protein and the membrane lipids [83].

The liposome flotation assay was also exploited by Vogt and coworkers to study how the group-specific antigen (gag) protein can sense the composition of the hydrophobic part of the bilayer during the process that leads to the viral assembly at the plasma membrane in the case of *Human immunodeficiency virus 1* (HIV-1) viral infection [84]. The obtained results showed that gag is sensitive both to the acyl chains of phosphatidylserine and to cholesterol concentration. Other details about the membrane environment allowed for the understanding of how gag recognizes membrane rafts in terms of their lipid composition.

The ability of liposomes to act as promoters for the aggregation of nanoparticles has been exploited by Saito et al. to develop an assay for assessing the presence of Rubella virus (RuV) in biological fluids [85]. It consists of a protocol for liposome flotation assays based on the set-up of direct interactions between RuV particles and the liposomal membrane components. Upon the application of density gradient fractionation, the RuV particles bound to liposomes shift to lower density fractions with respect to the unbound virus.

In the field of virus detection, in 1995, Reichert et al. developed sialic acid-conjugated liposomes mimicking the cell surface molecular recognition path occurring through the binding between sialic acid and hemagglutinin lectin that is present on viral surfaces [86]. Remarkably, the binding to influenza virus is accompanied by a liposome color change, from blue to pink/orange. This method is characterized by a LOD of influenza virus particles of ~1 HAU in 250 µL (~4000 viruses per µL). Using a method based on surface plasmon resonance and immobilized sialic acid containing liposomes, Hidari et al. reported LODs as low as ≈0.1 pM (≈6 × 10^4^ viruses per µL) [87]. In this context, it is worth referring to the work of Egashira et al., who developed and tested a detection method based on the combination of electrochemiluminescence with an immunoliposome-encapsulated rubidium complex [88]. The high sensitivity of the method allowed for the detection of hemagglutinin concentrations in influenza virus in a range as low as 3 × 10^−13^ to 4 × 10^−11^ g/mL, suggesting that 6 × 10^−19^ mol/50 µL could be considered as the LOD of this method for viral hemagglutinin. These results showed that the method owns a detection sensitivity that is in the attomolar level for detecting trace amounts of proteins in influenza virus.

An interesting liposome-involved approach to a semiquantitative test based on lateral flow assays (LFA) has been proposed by Baummer and coworkers [89]. As for common LFAs, the signal generation is provided by the use of colloidal gold particles that generate colored bands that report about the concentration of the analyte of interest. They used dye-loaded liposomes to pursue an enhanced signal response in a sandwich immunoassay for the detection of myoglobin in whole blood. The release of myoglobin in circulation is considered an important biomarker of a recent heart attack. In the assay, streptavidin-conjugated visible dye (sulforhodamine-B)-encapsulating liposomes could recognize a detector biotinylated monoclonal antibody against myoglobin.

Liposome immunoassays have also been applied in the analysis of foodstuffs through the development of systems capable of tagging with specific antibodies against the target pathogen. *Escherichia coli* and *Salmonella* have received considerable attention. It was shown by Chen and Durst that universal G protein liposomal nanovesicles could be easily conjugated by the antibodies within 30 min, and these conjugates (proteinG-immunoliposomes) showed a great detection ability against *E. coli*, *Salmonella*, and *Listeria* [90].

An interesting approach was developed by Zhao et al., who reported the use of liposome-doped nanocomposites as an artificial cell-based biosensor for the sensitive detection of a pore-forming emolysin, listeriolysin-O (LLO), from bacterial origin [91]. During pore formation and membrane insertion by LLO, the immobilized liposomes acted as cellular surrogates. Fluorescence quenching and leaching assays were used for measuring the integrity of liposomes in solid and solid-gel glass states.

In the field of pathogen detection, the work of Bui et al. is noteworthy, as they developed a liposome-amplified plasmonic immunoassay for the visual detection of single-digit-detectionof live pathogens, including *Salmonella*, *Listeria*, and *E. coli* O157:H7, in water and food samples [92]. Their work relied on the integration of cysteine-loaded nanoliposomes into a conventional ELISA as a signal amplifier as well as the release of cysteine from nanoliposomes to cause aggregation of plasmonic GNPs as a signal-amplified response. The lowest analyte concentration analyzed and detected with a visible color shift was found to be 6.7 attomolar, which is the lowest naked eye LOD reported without use of enzymes or visualization equipment.

Additionally, the cell-like appearance of liposomes makes them appropriate simulated cell models for studying and predicting the interactions between biologically active compounds and cell membranes, i.e., liposomes labeled with the water-soluble dye sulforhodamine B, interacting with influenza virus through the hemagglutinin pathway or the liposome blood–brain barrier and neural cell mimicking in the study of amyloid beta protein interactions [93,94].

Finally, it is worth noting that negatively charged vesicles and micro-organisms can be labelled, via simple electrostatic interaction, with cationic liposomes. This approach has been recently applied for the labeling of Gram-negative bacteria [95].

## 9. Conclusions

The use of liposomes in analytical methodologies continues to be under intense scrutiny in a wide array of applications. Many assays rely on their signal amplification power, with the aim of detecting analytes at low concentrations with high sensitivity. Liposomes are most commonly exploited as optical sensors, but it has been shown that they also work well as magnetic and electrochemical sensors. Overcoming the non-specific signal limitations due to undesired interactions of the lipidic nanoparticles, one may expect that even higher specificity and sensitivity in future liposome-based analytical assays could be achieved. Improving these materials for the detection of analytes in biofluids may open the possibility to develop fast and easy analysis for diagnostic purposes. The possibility of exploring new routes on the basis of the analogy liposomes have with whole cells appears analogously important. In principle, one may envisage the possibility of transferring the complex recognition pathways shown by cells to suitably functionalized liposomes, thus implementing artificial systems endowed with enhanced binding affinity with the biomarkers of interest thanks to the set-up of an improved thermodynamic frame. A variety of approaches have already been reported and one may foresee that the design of properly decorated liposomes will allow for the tackling of the highly specific recognition of whole cells, viruses, bacteria, and exosomes for the generation of an innovative diagnostic wave.

## Figures and Tables

**Table 1:** Scheme of liposome classification.

Vesicle Typology		Size	Number of Layers
Small Unilamellar	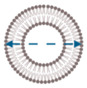		
Vesicle		20–100 nm	Single
(SUV)			
Large Unilamellar	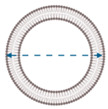		
Vesicle		100 nm–1 µm	Single
(LUV)			
Large Multilamellar	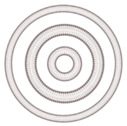		
Vesicle		>500 nm	Multiple, concentric
(MLV)			
Oligolamellar (Multivesicular)	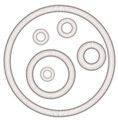		
Vesicle		100 nm–1 µm	Multiple, not concentric
(OLV)			
Giant Unilamellar	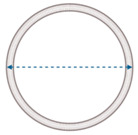		
Vesicle		>1 µm	Single
(GUV)			
